# Supplementary material for: Improvement of Luminescence Properties of Eulytite Single-Phase White Emitting Ca3Bi (PO4)3: Ce3+/Dy3+ Phosphor
Source: Molecules. 2023 Jun 24;28(13):4967. doi: 10.3390/molecules28134967 (PMC10343604; doi:10.3390/molecules28134967)
Supplement: Supplementary file 1 [file molecules-28-04967-s001.zip › molecules-2434356-supplementary.pdf]

Supplementary Materials

Improvement of Luminescence Properties of Eulytite Single-Phase White Emitting $Ca_3Bi(PO_4)_3$: Ce^{3+}/Dy^{3+} Phosphor

Mengjiao Xu, Jiamin Liang, Luxiang Wang *, Nannan Guo and Lili Ai

State Key Laboratory of Chemistry and Utilization of Carbon Based Energy Resources; College of Chemistry, Xinjiang University, Urumqi 830017, China; xmj_1117@163.com (M.X.); 19882996286@163.com (J.L.); guonan067@163.com (N.G.); ailili0709@163.com (L.A.)
* Correspondence: wangluxiangxju@163.com

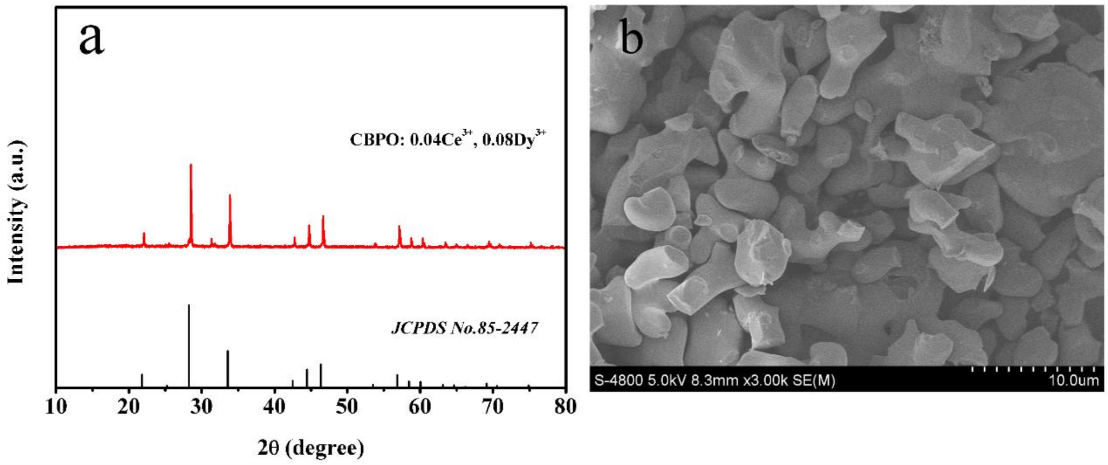

Figure S1. The XRD (a) and SEM (b) of the CBPO: $0.04Ce^{3+}$, $0.08Dy^{3+}$ sample after the working temperature of 473K.
